# Impact of COVID-19 on community mental health care referrals

**DOI:** 10.1192/j.eurpsy.2024.1055

**Published:** 2024-08-27

**Authors:** T. C. Gomes

**Affiliations:** Psychiatry, Tallaght university hospital, Dublin, Ireland

## Abstract

**Introduction:**

As the global community grapples with the aftermath of the COVID-19 pandemic, its reverberations extend beyond the realm of physical health, significantly impacting mental health care systems. This article delves into the multifaceted effects of COVID-19 on community mental health care referrals, scrutinizing the challenges, adaptations, and potential innovations that have emerged in the wake of this unprecedented crisis. By examining the nuanced interplay between the pandemic and mental health care access, we seek to shed light on crucial considerations for the future of community mental health services in a post-pandemic landscape.

**Objectives:**

To understand impact of Covid 19 pandemic on number of referrals received by a specific community mental health service.

**Methods:**

We analysed number of referrals to a specific community mental health services since July 2019 until July 2022.

**Results:**

During the period assessed we noticed a significant decrease to number of new referrals to a specific community mental health service with onset of covid 19 pandemic. We also noticed a progressive increase to the number of referrals in the first six months of July 2022.

**Conclusions:**

The COVID-19 pandemic has had a significant impact on attendance to healthcare appointments, leading to decreased attendance, shift to telemedicine, delays in care, increased no-shows, and rescheduling of appointments. The pandemic has also highlighted the importance of being prepared for and able to adapt to changes in the healthcare landscape.

**Disclosure of Interest:**

None Declared

